# Development and Optimization of a Multiplex Real-Time RT-PCR to Detect SARS-CoV-2 in Human Samples

**DOI:** 10.1155/2024/4894004

**Published:** 2024-03-11

**Authors:** Camilo Castellar-Mendoza, María-Angélica Calderón-Peláez, Jaime E. Castellanos, Myriam L. Velandia-Romero, Carolina Coronel-Ruiz, Sigrid Camacho-Ortega, Lilia J. Bernal-Cepeda, Lady López-Ibarra, Jhann A. Arturo, Félix G. Delgado, Hernando Gutierrez-Barbosa, Sonia Bohorquez-Avila, Johanna Madroñero, Zayda L. Corredor-Rozo, Sandra J. Perdomo-Lara, Angela Fonseca-Benitez, Eliana Calvo

**Affiliations:** ^1^Virology Group, Universidad El Bosque, Bogotá 110121, Colombia; ^2^Facultad de Odontología, Universidad Nacional de Colombia, Bogotá, Colombia; ^3^Inmugen Corporation, Bogotá, Colombia; ^4^Universidad El Bosque, Bogotá, Colombia

## Abstract

PCR and its variants (RT-PCR and qRT-PCR) are valuable and innovative molecular techniques for studying nucleic acids. qPCR has proven to be highly sensitive, efficient, and reproducible, generating reliable results that are easy to analyze. During the COVID-19 pandemic, qPCR became the gold standard technique for detecting the SARS-CoV-2 virus that allowed to confirm the infection event, and those asymptomatic ones, and thus save millions of lives. In-house multiplex qPCR tests were developed worldwide to detect different viral targets and ensure results, follow the infections, and favor the containment of a pandemic. Here, we present the detailed fundamentals of the qPCR technique based on fluorogenic probes and processes to develop and optimize a successful multiplex RT-qPCR test for detecting SARS-CoV-2 that could be used to diagnose COVID-19 accurately.

## 1. Introduction

In late 2019, a new Betacoronavirus was identified in humans, causing a severe pneumonia disease known as COVID-19 [1]. Since its emergence, the virus has infected almost 700 million people and caused over 6.5 million deaths worldwide. The excess mortality associated with the pandemic was estimated to be 15 million between 2020 and 2021. The rapid spread of the virus and its high mortality rate have created an urgent need to control viral transmission. However, the transmission of the virus from asymptomatic individuals has made it challenging to trace SARS-CoV-2 based solely on clinical symptoms. This highlights the need to implement rapid and specific techniques to efficiently detect SARS-CoV-2. Molecular tests such as reverse transcription-polymerase chain reaction (RT-PCR) and its quantitative variant RT-qPCR are excellent diagnostic options, given their ability to detect target nucleic acids with high sensitivity [2] (Figure S1).

Multiplex PCR is a technique that involves amplifying and detecting two or more gene sequences in the same reaction, using more than one primer pair in the amplification tube [3]. Some of the benefits of this technique include higher throughput (potentially more samples analyzed per plate), lower sample usage, and lower reagent usage, depending on the number of targets in the experiment, reducing the time and cost of analysis [4]. The development of PCR detection equipment with simultaneous multitarget detection and advances in probe chemistry have made comparative analyses standard in many areas of research and testing [5].

The qPCR based in probe hydrolysis is one of the most important tools for diagnosing and studying the SARS-CoV-2 due to its high sensitivity, specificity, and reliability as it depends upon the primer binding to its specific target sequences and fluorescent-based quantitative PCR assays to allow sensitive detection (Figure S2). Additionally, the one-step RT-qPCR became the preferred method over the two-step method owing to it being fast and efficient and involving limited sample handling, minimal experimental errors, and reduced bench time, allowing high-throughput testing necessary for the pandemic control [6]. Another advantage is the capability of hydrolysis probes in qPCR to detect multiple genes in a single reaction, which was important in a context of shortage of enzymes, probes, and plastic ware and the rising cost of enzymes and dual-labeled probes, due to the enormous demand for assays. Additionally, the hydrolysis probe qPCR allows to quantify and compare transcripts between control and experimental samples, to evaluate changes in gene expression (Figure S3). Low-income countries were the most affected by that limitation. This paper presents the fundamentals and principles of qPCR (in the Supplementary File) to better understand the technique, and additionally we describe the development of a multiplex RT-qPCR system for detecting SARS-CoV-2 virus during the onset and evolution of the COVID-19 pandemic in Colombia that allows the simultaneous detection of two viral genes and a human internal control for a rapid and specific diagnosis of SARS-CoV-2, obtaining a procedure sensitive, rapid, and accessible to track the pandemic virus.

## 2. Materials and Methods

### 2.1. Clinical Samples and RNA Extraction

In this study, 155 RNA samples obtained from nasopharyngeal swab samples of respiratory symptomatic patients with clinical suspicion of SARS-CoV-2 infection were selected and collected by a private diagnostic laboratory (Approval by Ethics Committee UEB-560-2020). These samples were previously evaluated using the commercial GeneFinder® COVID-19 Plus RealAmp RT-PCR kit, and 79 negative and 76 positive samples were confirmed. RNA extraction from clinical samples was performed by an in-house method using SpeedBead Magnetic Carboxylate Modified Particles and lysis buffer with guanidine salts developed and standardized at the Virology Laboratory of Universidad El Bosque. The elution volume used for this method was 50 µL.

### 2.2. Primers and Probes and *In Silico* Evaluation

**Figure 1:**
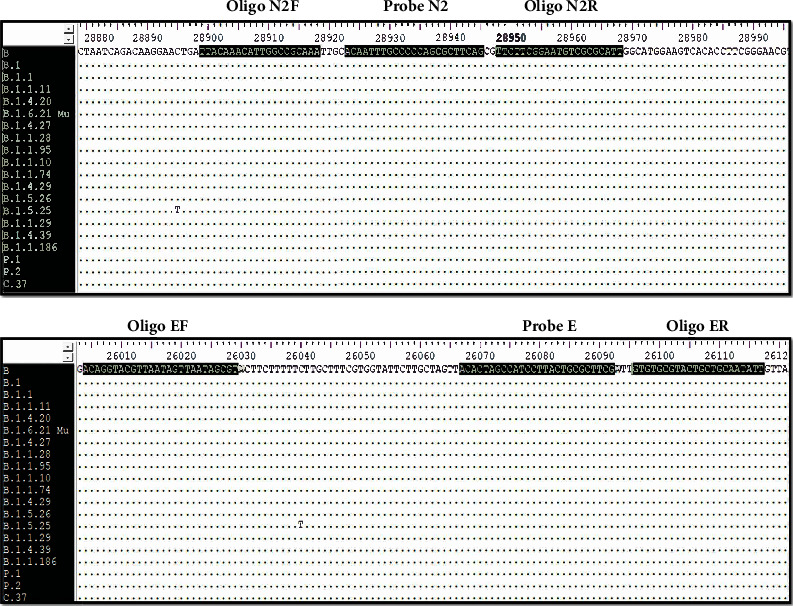
Multiple sequence alignment. Primers and probes for SARS-CoV-2 used in the multiplex reaction are highlighted at the first sequence. Each dot indicates a match or identity between the analyzed sequences. Main strains and variants circulating in Colombia were compared.

Primers and probes for E and N viral genes published by Corman et al. in the Charité University protocol were used [7]. The RNase P gene was used as a human internal control to assess the presence of amplification-susceptible RNA in the samples [7]. The probe for the E gene was labeled with FAM fluorophore, the N probe with Texas Red, and RNase P with HEX, and their respective quenchers. *In silico* evaluation of primers and probes included in the multiplex system was performed using 20 Colombian genome sequences reported in the GISAID database, with each sequence representing one of the lineages circulating in the country from 2020 to December 2021, including the Mu variant described in Colombia and other variants of interest in the region such as P1 and P2 in Brazil and C37 in Peru. The results showed that all primers and probes presented a 100% identity with those SARS-CoV-2 lineages (Figure 1). During first steps of standardization, amplicons were cloned and sequenced to confirm their identity.

### 2.3. RT-qPCR Assays for the Detection of SARS-CoV-2 and Single vs. Multiplex Performance Comparison

Reverse transcription and amplification of the viral genome were performed using the Luna® Universal One-Step RT-qPCR enzyme kit. The E and N viral genes were amplified individually (single reaction) and in combination with the RNase P gene (triplex reaction). Several concentrations of primers and probes were tested and finally selected at 0.2 *μ*M for both reactions (single and multiplex). Samples were considered positive when fluorescence exceeded the detection threshold at Cq less than 37 and a gradual increase during the amplification cycle, generating a typical sigmoidal amplification curve. Samples with Cq values greater than 37 were considered negative. The study also compared the performance of the E and N single reactions with that of the triplex reaction (E, N, and RNAse P) using a commercial SARS-CoV-2 RNA control, diluted to obtain concentrations of 100, 50, and 20 viral copies/*μ*L, and for the latter, serial two-fold dilutions were made to obtain concentrations of 10 and 5 viral copies/*μ*L, using nuclease-free water as the diluent. The single reactions were performed in duplicate and six replicates of the triplex reaction were performed to obtain the analytical sensitivity (LoD). Finally, to evaluate the performance of triplex RT-qPCR in clinical samples, 155 RNA samples from patients with suspected COVID-19 were selected and analyzed in a CFX-96 thermal cycler.

Calculations of sensitivity and specificity were performed by a classical approach, counting those samples positive that were obtained from confirmed SARS-CoV-2 patients (true positives) and negative samples that were from discarded cases (true negatives). We use the commercial and validated system GeneFinder Plus RealAmp Kit to compare the results in concordance with the developed RT-qPCR system described in the article. The reported performance of the commercial kit was sensitivity of 100% (95% CI: 88.6–100%) and specificity of 100% (95% CI: 88.6–100%).

## 3. Results

### 3.1. Evaluation of the Analytical Sensitivity of the Single E and N and E–N–RNAseP P Multiplex Reactions

**Figure 2:**
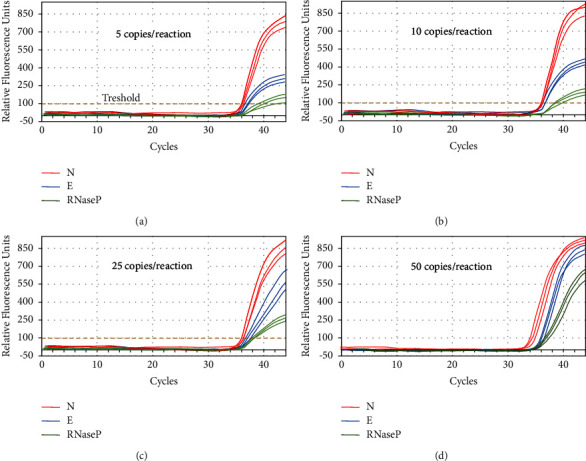
Amplification curves of different numbers of SARS-CoV-2 genome copies to evaluate the limit of detection (LoD). (a) Detection of 1 viral copy/*μ*L (5 copies per reaction). (b) Detection of 2 viral copies/*μ*L (10 copies per reaction). (c) Detection of 5 viral copies/*μ*L (25 copies per reaction). (d) Detection of 10 viral copies/*μ*L (50 copies per reaction). Reactions were performed in a BioRad CFX96 Thermocycler.

The study evaluated the analytical sensitivity of the single E and N qPCR and E–N–RNAse P multiplex reaction. The results showed no significant differences in the Cq and relative fluorescence units (*p*=0.1) between the single reactions of the E and N genes and the multiplex reaction (viral E–N in combination with RNAse P) (Figure 2). The mean and standard deviations of Cq were similar between groups at all dilutions, and the system reliably detected up to 2 viral copies/*μ*L, corresponding to 10 genomic viral copies per reaction.  Table 1 presents the data obtained after evaluation of limit of detection.

### 3.2. Evaluation of the Clinical Sensitivity and Specificity of the E–N–RNAse P Multiplex System

**Figure 3:**
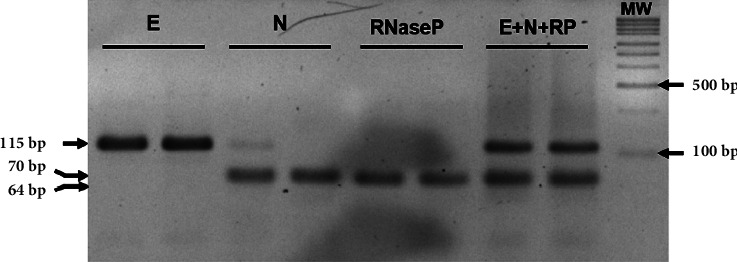
Separation of SARS-CoV-2 amplicons in a 3% agarose gel electrophoresis. Single and multiplex targets amplifications were performed.

The study evaluated the clinical sensitivity and specificity of the developed E–N–RNAse P multiplex system by testing 155 clinical samples and comparing the results with those obtained by the commercial GeneFinder® COVID-19 Plus RealAmp RT-PCR kit. The commercial kit detected three SARS-CoV-2 genes (E, N, and RNA-dependent RNA polymerase, RdRp) and confirmed 76 positive cases, and 73 out of these were also confirmed with the standardized multiplex system. Final analysis resulted in 96% specificity and 100% sensitivity regarding the commercial kit (Table 2). To verify that the amplification signal obtained in the RT-qPCR reaction corresponded to the expected products for each amplification target, the products obtained in the single reaction (E or N), duplex (E–N), and multiplex (E–N–RNAse P) were evaluated using 3% agarose gel electrophoresis. The bands corresponding to the fragments: E with a size of 115 bp, N of 70 bp, and the fragment corresponding to RNAse P of 64 bp, were observed (Figure 3). However, because of the closeness between the N and RNAse P amplicons, the three products of the E–N–RNase P reaction were not simultaneously observed.

## 4. Discussion

During the COVID-19 pandemic, RT-qPCR became an essential tool for managing and providing epidemiological information in all countries [8]. In Latin American countries, such as Colombia, this technique is well established, but mainly in research centers and laboratories belonging to universities. These became a support network for diagnosing SARS-CoV-2 infections in the Colombian health system. However, one of the challenges was access to commercial kits for in vitro diagnosis, as there was little availability in this region. Likewise, the price of these commercial kits exceeds USD $1600 (100 reactions), such as Allplex™ 2019-nCoV Assay100 (USD $1430), MolecuTech® Real-Time COVID-19 (USD $1565), and Dynamiker Novel Coronavirus 2019-nCoV (USD $1665), while the protocol developed and adapted in this article and used during the pandemic in Colombia costs USD $165 (100 reactions).

During most of 2020, only protocols developed by different CDCs in countries such as China and the United States and research institutes in European countries such as Germany and France were available [9, 10]. Therefore, research laboratories supported the diagnosis of COVID-19 using these open shared protocols. In this study, the protocols were adapted and optimized by adjusting the primer-probe sets for a single reaction for detecting two viral genes and a human control gene, which helped improve the diagnostic processes [11]. The study presents a rapid and simple method for developing and adapting a multiplex system for real-time PCR diagnostics with retrotranscription, using independently reported and evaluated primer-probe sets as a starting point. Using this specific protocol, our laboratory and others processed almost 100 000 samples during the first year of the pandemic.

The multiplex reaction did not individually affect the sensitivity and specificity reported by the protocols. The annealing temperature of the recommended primers and probes (58°C) was conserved, which contributed to the regulation of nonspecific amplification during the amplification protocol. The results showed that the multiplex system can discriminate between true positives and negatives, identical to a commercial kit (GeneFinder® COVID-19 Plus RealAmp RT-PCR), which shows a LoD of 0.5 cp/*μ*L. Adapting protocols to each laboratory and geographic region is essential to preserve the technical-scientific recommendations and optimize them for each context [12–14].

Latin America, despite contributing less than 10% of the world's population, has experienced one-third of COVID-19 cases and 25% of deaths worldwide. This is due in part to the economic and health system challenges faced by underdeveloped countries, as well as the weakness and asymmetry of molecular diagnosis laboratories and a global shortage of reagents and supplies, which explain the low rates of confirmatory tests done for each reported case in Latin America [15]. However, during the first year of the pandemic, many Latin American university research laboratories transferred their molecular skills to SARS-CoV-2 diagnosis, helping public health authorities to follow and control the virus transmission. For example, Peruvian academic and researchers standardized and validated a multiplex qPCR [16], while Ecuadorian and Uruguayan universities developed new qPCR systems that reached optimal clinical performance [17]. In Colombia and Brazil, university laboratories improved alternative low-cost multiplex PCR or Sybr green protocols to confirm positive samples [11, 18, 19]. The protocols mentioned above used the reported primers and probes tested until then and demonstrated optimal performance in terms of sensitivity and specificity. These efforts shed light on the conditions required to develop a molecular diagnosis assay and allowed for the gain of scientific and technical capabilities for public health surveillance. The developed protocols were important to cope with the sanitary crisis derived from the pandemic and to unblock the historic backlog in Latin American biotechnology.

This academic and technical work presented the hydrolysis probe qPCR fundamentals to spread the principles to scientists and diagnostic laboratories and resume the necessary steps to implement the technique for clinical diagnosis [20].

## 5. Conclusions

This paper presents the fundamentals and principles of quantitative real-time PCR (qPCR) as a learning tool in both academic and diagnostic environments (Supplement). Additionally, the study established a diagnostic method for SARS-CoV-2 using multiplex amplification and found that primers and probes were still adequate for the SARS-CoV-2 strains circulating in Colombia. The mixed preparation of reagents for detecting two viral genes and one human gene detected the virus from respiratory patients with high accuracy, specificity, and sensitivity, which showed low cost and high clinical performance compared with a commercial molecular system. The standardized protocol was useful in following the SARS-CoV-2 circulation in Colombia during the challenging pandemic when the shortage of reagents and lab ware was characteristic.

## Figures and Tables

**Table 1:** Cq (and standard deviation) obtained for different copy number of templates to evaluate the limit of detection (LoD).

Gene target	Copies per reaction							
	1		5		10		25	
	E	N	E	N	E	N	E	N
Replicas	18	18	18	18	18	18	18	18
Positive	7	12	11	18	18	18	18	18
Cq (mean)	40.64	39.79	39.35	36.83	36.63	35.54	34.60	33.25
SD	0.44	1.10	0.81	0.57	0.37	0.45	0.53	0.63

**Table 2:** Clinical sensitivity and specificity of the developed multiplex system.

Multiplex E/N/RP	Reference method: GeneFinder		
	Positive	Negative	Total
Positive	73	3	76
Negative	0	79	79
Total	73	82	155
Sensitivity	**100%**		
Specificity	**96%**		

The bold values are the sensitivity and specificity of the RT-qPCR in comparison with a commercial kit.

## Data Availability

The Biorad CFX-96 readings, raw data, and results of the clinical evaluation used to support the findings of this study have been deposited in the Mendeley Data repository (DOI: 10.17632/dxfs9yps45.2).

## Supplementary Materials

Supplementary Materials The supplementary file presents in-depth principles of PCR and quantitative PCR, reviewing the fundamentals of amplification, type of chemistries involved in fluorescence signals, and quantification strategies using real-time PCR.
