# Supplementary material for: Development and Optimization of a Multiplex Real-Time RT-PCR to Detect SARS-CoV-2 in Human Samples
Source: Int J Microbiol. 2024 Mar 11;2024:4894004. doi: 10.1155/2024/4894004 (PMC10948217; doi:10.1155/2024/4894004)
Supplement: Supplementary Materials — The supplementary file presents in-depth principles of PCR and quantitative PCR, reviewing the fundamentals of amplification, type of chemistries involved in fluorescence signals, and quantification strategies using real-time PCR. [file 4894004.f1.zip › 19022024 RT-qPCR Supplementary File Word.docx]

# Supplementary File

# International Journal of Microbiology

By the end of 2019, a new *Betacoronavirus* had been detected in humans. Like the related virus, the so-called SARS-CoV-2 caused a severe pneumonia disease known as COVID-19. There was an urgency to implement COVID-19 virus detection techniques and reverse transcription coupled to polymerase chain reaction (RT-PCR) were excellent options, given their ability to detect the sequence target with very high sensibility (<100 copies/mL) and specificity. This Supplementary file, aim to review the fundamentals of this technique for detection of virus genetic material.

Principles of the Technique**.**

Polymerase chain reaction (PCR) is a molecular technique that has had the most significant impact in the history of molecular biology. It was developed by Kary Mullis in 1983 and is based on a thermo-stable polymerase-dependent repetitive reaction that allows the generation of multiple copies identical to a specific section of DNA known as a template.

Each PCR assay requires the following:

-A small amount of DNA, which can be obtained from any tissue or body fluid such as blood, urine, and saliva, containing the target sequence to be amplified, called the DNA template.

-Two primers or oligonucleotides (one for each strand), short DNA segments of 16–30 nucleotides complementary to the template, flanking the region to be amplified. They provide the 3'OH end to the DNA polymerase to initiate elongation.

-Deoxynucleotide triphosphate or dNTPs (dATP, dCTP, dGTP, and dTTP) act as bricks and provide the energy necessary for DNA polymerization.

-A thermo-stable DNA polymerase such as *Taq* polymerase (extracted from *Thermus aquaticus*) catalyzes DNA elongation at high temperatures.

-Reaction Buffer avoid changes in the pH during the reaction; typically, it contains Tris-HCl, KCl, and MgCl_2_. The concentration of the latter usually has an essential effect on the specificity and yield of the reaction. A concentration between 1-4 mM is optimal.

The PCR reaction has three stages: **denaturation**, at temperatures above 84°C, the hydrogen bonds that stabilize double-stranded DNA break separating the DNA molecule into two single-strands. Once the DNA strands are separated, the primer hybridization or **annealing** phase begins, which takes place between 40°C–60°C. Lower temperatures allow hydrogen bonds formation and the DNA molecules to recover their double-stranded conformation. The higher the temperature, the more restrictive the primer annealing and, thus, the greater the specificity of the reaction. Finally, the **elongation** occurs at 72°C, where the synthesis of the complementary DNA strand occurs through the action of TaqPol. Thus, DNA template regions downstream of the primers are selectively synthesized (Figure S1).

**Figure S1. PCR process.**

The three steps mentioned earlier comprise one amplification cycle. Each cycle theoretically doubles the amount of the DNA, at the end, it will produce 2^n^ times the target fragment (where n=number of cycles).

Currently, there are more than 30 types of PCR with defined applications and protocols, but all are based on similar principles. For example, the RT-PCR uses RNA as the starting material; the complementary DNA (cDNA) is synthesized using oligo-dT, random primers or specific primers, and an RNA-dependent DNA polymerase derived from avian myeloblastosis virus (AMV-RT) or Moloney murine leukemia virus (MMLV) which makes the retrotranscription at a temperature between 37°C–42°C. Subsequently, the initial RNA is degraded, and double-stranded DNA (dsDNA) is produced and used as a template for PCR amplification. An advantage of this technique is that cDNA is not subject to degradation by RNases, which makes it more stable than RNA (10). Choosing the correct enzyme is essential, as each offers advantage. For example, AMV-RT can help eliminate problems associated with RNA secondary structures, whereas MMLV-RT allows the synthesis of long amplicons and may be a better choice for amplifying full-length cDNA molecules.

The advantages of PCR are its simple implementation, sensitivity, quick result production, and low cost compared to other molecular methods. Some limitations of end-point PCR are the possibility of cross-contamination and the requirement of gel electrophoresis to analyze the amplicon; results are not quantitative, just indicate presence or absence, according to the fragment size obtained.

To solve these problems, quantitative or real-time PCR (qPCR) was developed which is at least 100 times more sensitive than the end-point PCR (3), making it the chosen technique for gene expression analysis, pathogens detection, genetic diseases identification, treatment progress, among other applications.

**Real-Time PCR**. This strategy allows easy and reproducible quantification and faster results by eliminating the post-amplification step. The method is based on detecting a fluorescent signal produced in each cycle, which is proportional to the amount of PCR product generated. It requires a specialized thermal cycler capable of reading the fluorescence signal and software capable of analyzing the data.

As the reaction occurs, the raw data are presented as a plot of the number of cycles (Cq on the X-axis) versus fluorescence units (RFU on the Y- the axis) in which four phases are evident. In the baseline at the beginning of a reaction, the amount of PCR product is low, and the fluorescence signal does not differ from the background. During the exponential phase, the fluorescence signal increases significantly and rapidly above the background. The threshold cycle (Ct or Cq) defines the number of cycles before amplicons are detectable. Through the linear phase, the product amount doubles each cycle. At the end of the exponential stage, the plateau is reached the products are no longer duplicated; therefore, the fluorescence signal does not increase. The plateau occurs when the substrates are exhausted, and the DNA polymerase is at the end of its lifetime (Figure S2).

Quantification is possible due to the fluorescence signal read cycle by cycle. There are two types of chemistry: the first is based on dsDNA-intercalating molecules, and the second is based on fluorochrome-labeled probes.

**Figure S2. Quantitative or Real time PCR.**

SYBR® Green I and Other dsDNA Intercalants.

Small molecules that bind to dsDNA can be classified into intercalants and minor groove binders (MGB). Ethidium bromide is an example of an intercalant, whereas Hoechst 33258 is an example of an MGB (12). These molecules should exhibit an increase in fluorescence when bound to dsDNA and should not cause PCR inhibition. The most commonly used is SYBR^®^ Green I, an asymmetric cyanine dye with two positive charges that, under standard PCR reaction conditions, contributes to its high binding affinity to dsDNA.

In addition to SYBR® Green I, various fluorescent DNA intercalants are currently available, such as ethidium bromide, YO-PRO-1, SYBR^®^ Gold, SYTO, BEBO, BOXTO, and EvaGreen. The latter is a third-generation intercalant with several advantages, such as lower PCR inhibition than that of SYBR^®^ Green I. It can be used under saturated conditions to generate more intense fluorescent signals.

These molecules intercalate all the dsDNA sequences present in a reaction, including nonspecific products and primer dimers; therefore, melt curve analysis is necessary to check the specificity of the reaction (Figure 2B). Molecules of dsDNA of different lengths and base compositions dissociate into single strands at different temperatures; the melting temperature (Tm) is defined as the temperature at which 50% of the dsDNA molecules are separated. At the end of the amplification cycles, a gradual and slow rise in temperature from 65 to 95°C causes dsDNA dissociation. The intercalants are no longer associated, resulting in a loss of the fluorescence signal, which is recorded and transformed as the melting curve. This plot should display a single sharp peak since each amplicon in the sample will generate a different curve.

Fluorophore-labeled Oligonucleotides.

Oligonucleotides bound to fluorophores are used in qPCR as specific detection probes designed to bind exclusively to the target sequence and can distinguish products that differ by a single base. Primer probes and probes employ a Förster resonance energy transfer (FRET) interaction, which involves the interaction of two types of fluorophores: donors or reporters and acceptors or quenchers nearby. The donor's emission spectrum must overlap the absorption spectrum of the acceptor; thus, when the donor molecule is excited, the energy absorbed is transferred to the acceptor fluorophore and extinguished (quenched). When the fluorophores are separated, FRET is disrupted, resulting in a fluorescent signal (Figure S2C).

Unlike dsDNA intercalants, hydrolysis probes (TaqMan) hybridize to a specific sequence located in the region flanked by the primers (Figure S2E). The probe must exhibit higher Tm than the primers (typically 8°C–10°C), hybridizing to the template before the primers to their respective sites. During the extension step, the probe is hydrolyzed by the 5′–3′ exonuclease activity of Taq polymerase, releasing the donor from the quencher influence and generating a fluorescence signal (Figure S2C). The latest generation of TaqMan probes carries an MGB molecule at their 3′ end, which selectively binds non-covalently to the minor groove of DNA. Consequently, Tm increases, resulting in higher sensitivity and resolution.

One of the main advantages of using probes in qPCR is that multiple targets (currently, up to five) can be detected simultaneously in a single reaction tube. Multiplex PCR reduces reagent and material consumption, analysis time, and variability in the results. However, it is necessary to ensure good sensitivity, specificity, and efficiency for each target.

**Quantification strategies**

There are two strategies for the template quantification: absolute and relative. Absolute quantification calculates the copy number of the target sequence in a sample using a calibration curve constructed with tenfold serially diluted standards of known concentration. There is a linear relationship between Cq and the logarithm of the starting DNA amount (Figure S3). The line's correlation coefficient (R^2^) should be 0.99 or greater and cover the experimental samples' range of interest.

The slope of a standard curve is commonly used to estimate the PCR amplification efficiency (E= -1+10^(-1/slope)^); values between -3.58 – -3.10 correspond to efficiencies between 90 % – 110%. Ideally, it should be -3.32, indicating that the template was duplicated after each cycle. Efficiencies <90 % suggest bad primer design, inadequate annealing temperature, non-optimal reagent concentrations, or PCR inhibitors. Efficiencies >110 % may be due to primer-dimers or non-specific products (when using intercalating dyes).

**Figure S3. Real Time Quantification strategies.**

The relative quantification compares the gene expression in one sample to another, using at least one reference gene and one sample control or calibrator. Results are expressed as a fold change in the target expression relative to a calibrator sample. The ∆∆CT method is one of the most used to calculate differences between samples; however, it is based on normalization with a single reference gene, and the target and reference gene must be amplified with comparable efficiencies. Mathematical models that include normalization using multiple reference genes and the efficiency calculated for each sample allow more confident results than 2^-∆∆Ct^ method.
