# Supplementary material for: Development and Optimization of a Multiplex Real-Time RT-PCR to Detect SARS-CoV-2 in Human Samples
Source: Int J Microbiol. 2024 Mar 11;2024:4894004. doi: 10.1155/2024/4894004 (PMC10948217; doi:10.1155/2024/4894004)
Supplement: Supplementary Materials — The supplementary file presents in-depth principles of PCR and quantitative PCR, reviewing the fundamentals of amplification, type of chemistries involved in fluorescence signals, and quantification strategies using real-time PCR. [file 4894004.f1.zip › Figure Suppl 1.pptx]

## Slide 1
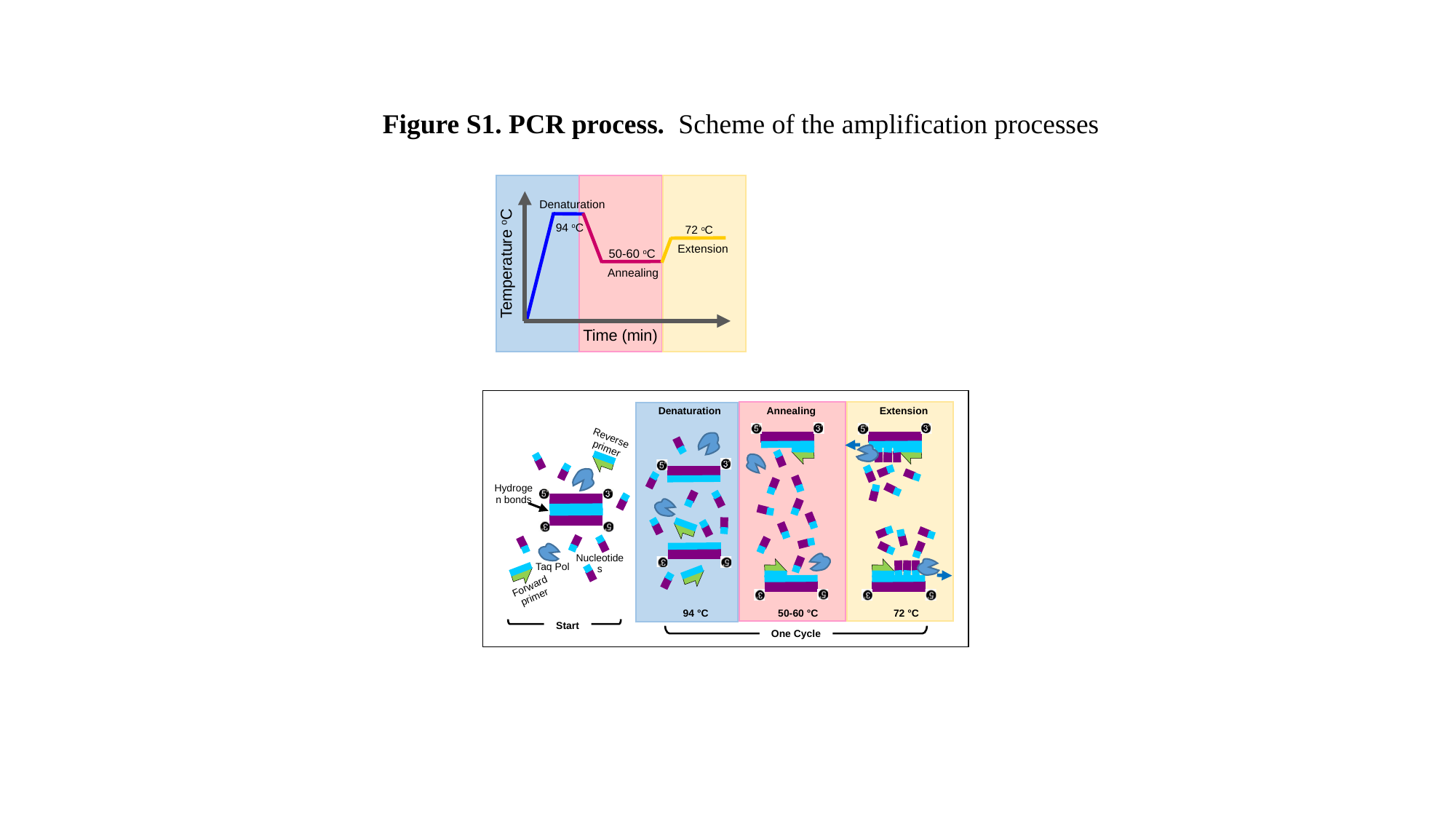

Figure S1. PCR process. Scheme of the amplification processes
Denaturation
94 oC
72 oC
Extension
50-60 oC
Temperature oC
Annealing
Time (min)
Denaturation Annealing Extension
Reverse primer
Hydrogen bonds
Nucleotides
Taq Pol
Forward primer
94 °C 50-60 °C 72 °C
Start
One Cycle
