# Supplementary material for: Development and Optimization of a Multiplex Real-Time RT-PCR to Detect SARS-CoV-2 in Human Samples
Source: Int J Microbiol. 2024 Mar 11;2024:4894004. doi: 10.1155/2024/4894004 (PMC10948217; doi:10.1155/2024/4894004)
Supplement: Supplementary Materials — The supplementary file presents in-depth principles of PCR and quantitative PCR, reviewing the fundamentals of amplification, type of chemistries involved in fluorescence signals, and quantification strategies using real-time PCR. [file 4894004.f1.zip › Figure Suppl 2.pptx]

## Slide 1
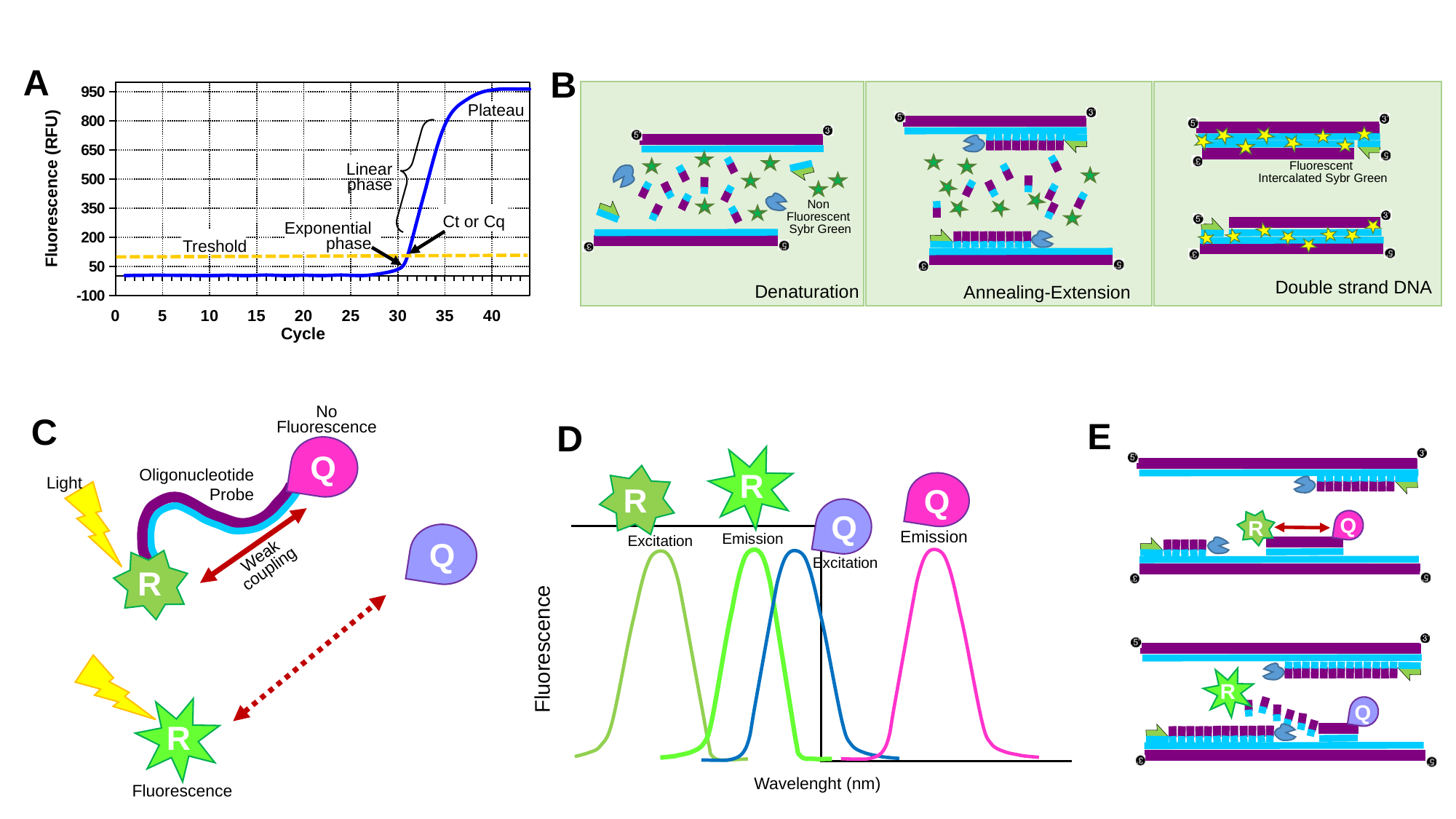

A
B
### Chart
| Category | RFU | |
|---|---|---|
Plateau
Fluorescent
Intercalated Sybr Green
Linear phase
Fluorescence (RFU)
Non
Fluorescent
Sybr Green
Ct or Cq
Exponential phase
Treshold
Double strand DNA
Denaturation
Annealing-Extension
Cycle
No
Fluorescence
C
E
D
Q
Oligonucleotide Probe
R
Light
R
Q
Q
Q
R
Emission
Emission
Q
Excitation
Weak coupling
Excitation
R
Fluorescence
R
Q
R
Wavelenght (nm)
Fluorescence
