# Supplementary material for: Development and Optimization of a Multiplex Real-Time RT-PCR to Detect SARS-CoV-2 in Human Samples
Source: Int J Microbiol. 2024 Mar 11;2024:4894004. doi: 10.1155/2024/4894004 (PMC10948217; doi:10.1155/2024/4894004)
Supplement: Supplementary Materials — The supplementary file presents in-depth principles of PCR and quantitative PCR, reviewing the fundamentals of amplification, type of chemistries involved in fluorescence signals, and quantification strategies using real-time PCR. [file 4894004.f1.zip › Figure Suppl 3 Final.pptx]

## Slide 1
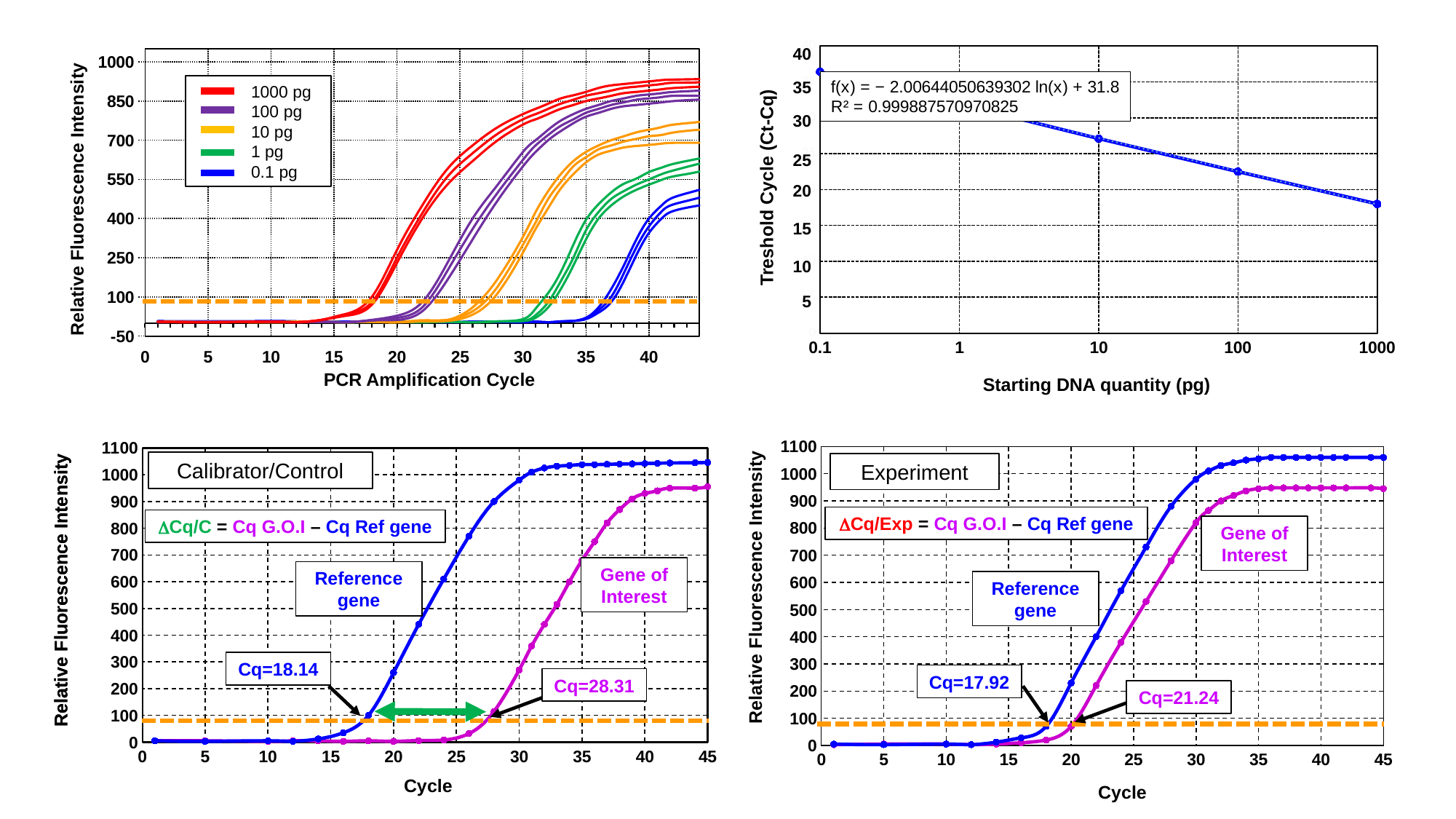

### Chart
| Category | |
|---|---|
### Chart
| Category | | | | | | | | | | | | | | | |
|---|---|---|---|---|---|---|---|---|---|---|---|---|---|---|---|40
35
30
25
20
15
10
5
1000 pg
100 pg
10 pg
1 pg
0.1 pg
Treshold Cycle (Ct-Cq)
Relative Fluorescence Intensity
A
PCR Amplification Cycle
Starting DNA quantity (pg)
### Chart
| Category | | | | |
|---|---|---|---|---|
### Chart
| Category | | |
|---|---|---|Calibrator/Control
Experiment
DCq/Exp = Cq G.O.I – Cq Ref gene
DCq/C = Cq G.O.I – Cq Ref gene
Gene of Interest
Gene of Interest
Reference gene
Relative Fluorescence Intensity
Reference gene
Relative Fluorescence Intensity
Relative Fluorescence Intensity
Cq=18.14
Cq=17.92
Cq=28.31
Cq=21.24
Cycle
Cycle
